# The Normative Orientations of Climate Scientists

**DOI:** 10.1007/s11948-014-9605-1

**Published:** 2014-11-08

**Authors:** Dennis Bray, Hans von Storch

**Affiliations:** Helmholtz Zentrum Geesthact, Institute for Coastal Research, 21502 Geesthacht, Germany

**Keywords:** Science ethics, Climate science, Merton’s CUDOs, Climate scientists

## Abstract

In 1942 Robert K. Merton tried to demonstrate the structure of the normative system of science by specifying the norms that characterized it. The norms were assigned the abbreviation CUDOs: Communism, Universalism, Disinterestedness, and Organized skepticism. Using the results of an on-line survey of climate scientists concerning the norms of science, this paper explores the climate scientists’ subscription to these norms. The data suggests that while Merton’s CUDOs remain the overall guiding moral principles, they are not fully endorsed or present in the conduct of climate scientists: there is a tendency to withhold results until publication, there is the intention of maintaining property rights, there is external influence defining research and the tendency to assign the significance of authored work according to the status of the author rather than content of the paper. These are contrary to the norms of science as proposed by Robert K. Merton.

## Introduction

“although the ethos of science has not been codified, it can be inferred from the moral consensus of scientists as expressed in use and wont, in countless writings on the ‘scientific spirit’ and in moral indignation directed toward contraventions of the ethos”.

(Merton 1942:117).

Climate change has evolved to be a public and political issue that is morally charged, value laden and discussed in terms of global welfare, issues well beyond the expertise of climate scientists. Debates concerning climate change emanate from scientific expertise and non-expert sources alike. With the future of the world as we know it claimed to be at stake, as is the case with climate change, and the changes deemed necessary, or not necessary, to prepare for or to combat the change, science, policy and the public no longer debate separately and the norms of science could be expected to change considerably when science enters the public and policy realms. All of this is common when “post-normal” conditions prevail (Funtowicz and Ravetz 1985; van der Sluijs 2010), and climate science was found to be in such a regime (Bray and von Storch 2010; Krauss and von Storch 2012). Post-normal circumstances, as described by Funtowicz and Ravetz, point to the likelihood that knowledge from science intersects with strongly held values.

This, in itself, is not unique. Genetic sciences and technology and, to some degree, aspects of pharmacological sciences, have been, and continue to be, debated in a similar manner as climate change science, although typically with less notoriety and less internal conflict. Furthermore, while these sciences also generate moral concerns, they also have a considerable interest in monetary profits. Climate change science, however, has no direct monetary incentive; it deals with a different currency. Its motivation is limited to the production of knowledge and a moral imperative, making it somewhat different from for-profit science, and indeed, from classical explorations of knowledge-for-knowledge’s-sake. The distinction is also somewhat apparent in the sponsors of the research. Furthermore, the study of climate change does not normally result in the registration of patents. There are, of course, other disciplines that share a similarity to climate change in terms of the production of knowledge but typically they are not so entwined with political policy and do not investigate omnipresent matters. Finally, rarely do other scientific endeavors address the future more than the past.

The intention of this paper is to assess the perceptions of the norms of science as held by climate scientists and to, as well as possible, determine if these have an impact on the normative structure of the science. Norms are collective expectations of what constitute right or wrong behavior. Norms pertain to the structure of ideologies. They are ideal abstractions, not elements of daily praxis. According to Durkheim (1995) the significance of a norm is indicated by the extent of moral outrage or indignation that ensues when a norm is violated. Robert K. Merton (1942) presented what he thought to be the basic norms of science, now often referred to as CUDOs, in ‘The Social Structure of Science’. While Merton’s CUDOs left a long legacy of debate, and are now perhaps considered somewhat outdated, and are not as likely to have any influence on the daily practice of science, the framework they present remains important in the discussion of the production of knowledge and change in the social structure of science.

Merton (1942:118) states ‘the mores of science possess a methodological rationale but they are bonding, not because they are procedurally efficient, but because they are believed right and good. They are moral, not technical prescriptions.’ Merton tried to demonstrate the structure of the normative system of science by specifying the norms that characterized it. Mertonian norms consist of *C*ommunism, *U*niversalism, *D*isinterestedness, and *O*rganized *s*kepticism, commonly referred to as Merton’s CUDOs. Since their inception, Merton’s CUDOs have received both praise and criticism. Merton’s notion of CUDOs might now seem somewhat of an antiquated approach to address the normative structure of climate science but they still provide a convenient analytical framework for investigating the cultural norms of science. According to Kellogg (2006:6).

Yet though Merton’s particular form of analysis may seem outdated from one perspective, the norms he named in 1942 have persisted impressively in public understanding. We still tend to assume that science follows the Mertonian framework or would, if social factors did not keep getting in the way. *Of course* claims should be evaluated on their merit, not on who made them; *of course* scientific knowledge should be open to inspection and evaluation; *of course* personal interests should be subordinated to the scientific enterprise; *of course* the institutions of science should pursue rigorous testing of hypotheses. Such views are hardly controversial; they represent the conventional wisdom about what we think, or what we hope, science to be. (The status of such views as conventional wisdom helps explain the widespread resistance among scientists to strong claims by the sociology of science, which are taken as attacking the realization, if not the ambition, of scientific practice.)

(Original emphasis, Kellogg 2006:6).

Merton (1942) noted that deviations from the norms were also part of social structure of science. Mitroff (1974) provided empirical evidence of counter-norms, suggesting that there is not one set of norms operating in science, but at least two: for each of the norms identified by Merton, there is an opposing norm that justifies the opposite. Mulkay (1976) identified the counter-norms as Solitariness, Particularism, Interestedness and Dogmatism.

Ziman (2000:31) cautions, however, ‘[…] norms only affirm ideas; they do not describe realities.’ Nonetheless, as Cloitre and Shinn (1985:31) state, ‘The Mertonian model affirms that science and non-science are clearly demarcated.’ As Zuckermann (1977:123) puts is, ‘norms are, of course, not behavior. […] they are standards taken into account in behavior and standards by which actual behavior is judged.’

This analysis is not about how climate scientists behave but about how climate scientists think they should behave. It is not about how realistic these norms are in science in general (Stehr 1978). The paper deals only with climate science, which has recently seen claims (Brysse et al. 2013:327) that an alleged bias towards more conservative scientific claims stem from ‘adherence to the scientific norms of restraint, objectivity, skepticism, rationality, dispassion, and moderation’. Also, an analysis of the stolen “ClimateGate” e-mails by Grundmann (2012) pointed to practices which were inconsistent with Merton’s norms.

The interest here is to measure the moral prescriptions–norms and counter-norms–that constitute part of the social system of climate science. The levels of subscription will be compared to the work of Macfarlane and Chen (2008) and Anderson et al. (2010) where possible. It is well noted that there are difficulties with how to elicit observable expressions of these norms. Ziman (2000) points out ‘newcomers to research soon discover that they are not just learning technical skills. They are entering a self-perpetuating ‘tribe’ whereby their behavior is governed by many unspoken rules. In essence, they represent part of the ‘culture’ of science and as such share the common problems of studying culture, namely, that the scientists in question are so immersed in their culture that the operating normative system is invisible to them. […] [One] approach is to construct statements of behavior that fall under the rubric of normative principles and then measure scientists’ subscription to such behaviours. This approach clearly falls short of capturing complex norms, but instead provides some measure of behaviors that indicate or reference norms’, an approach used by Anderson et al. (2010), Anderson (1996), (2000) Anderson and Louis (1994), Louis et al. (1994), Louis et al. (1995) and (Ziman 2000:31).

Anderson et al. (2010) drew a sample from ‘lists of scientists supported by funding from the National Institutes of Health [USA]’. In their analysis they concluded that ‘subscription to the counternorms was much lower than for norms. [and] There are no clear patterns evident in the cross-disciplinary comparisons of the original norms and counternorms, except that the physics/mathematics/engineering group is at an extreme in each case: higher on the norm of communality and lower on the counternorms of particularism and self-interestedness among the early-career respondents, and higher on individualism in the mid-career group.’

Macfarlane and Chen (2008), using a multidisciplinary sample of academics, examined the relevance of Merton’s norms to contemporary academia in the UK. In their findings they reported that 21 % of the respondents from the ‘natural sciences’ agreed that they were secretive about their work in progress’ but that this was not statistically significant from other disciplines and secrecy was independent of discipline. Fifty-three percent of all respondents agreed ‘that their intellectual work was influenced by personal beliefs and values.’ Other conclusion reached include: ‘the norm of disinterestedness is perceived of as the least popular contemporary academic value’; 42 % linked their research with funding opportunities, and; 68 % (37 % for natural scientists) ‘supported the idea of academics expressing their views in public’. The norm of organized skepticism was omitted from the survey. Overall, Macfarlane and Chen (2008) drew the conclusion that there was substantial support for the norm of communism and a strong sense of the importance of protecting individual property rights.

In the following, focus is on a single group of scientists, namely climate scientists and what level of subscription to Mertonian norms exists in climate science. As best as possible, the results pertaining to climate scientists will be compared to the broader scientific community as represented in Anderson et al. (2010) and Macfarlane and Chen (2008).

## Materials and Methods: The Survey of Climate Scientists

To undertake an analysis of the norms of climate scientists, data was collected using an on-line survey; A Survey of the Perceptions of Climate Scientists 2013 (Bray and von storch 2014. Data and codebook are available at: https://www.academia.edu/7454421/CliSci_2013_Data_Set_Excel_Format_with_code_book).

The survey used a non-probability convenience sample. The sample consisted of a list of authors drawn from climate science journals with the 10 highest impact ratings between 1998 and 2008 (this list was used previously for a survey conducted in 2008; a list of authors who contributed to the Oreskes (2004) study; a list of member of climate research institutes drawn from institute websites (Bray and von Storch 2010); the IPCC list of contributors (2013) and the ClimList mailing list (2013). After removing duplicates, the possible number of respondents was 5,947. After removing non-valid email addresses the number of possible respondents was reduced to 4,491. There were 286 valid returns for a response rate of approximately 7 %.

Such response rates are not untypical for on-line surveys. As a general comment on sampling and response rates, sampling special groups (scientists) often results in a comparatively difficult sample selection and a comparatively low response rate. The difficulty of selecting such a sample is discussed in Committee on Assessing Fundamental Attitudes of Life Scientists as a Basis for Biosecurity Education, National Research Council (2009) report ‘A Survey of Attitudes and Actions on Dual Use Research in Life Sciences’. Here the target population was US life scientists. The report notes no complete list of the population was available or even known. The alternative chosen was to find a sample through the use of professional societies.

Response rates for mail out hard copy surveys and on-line surveys also differ, with response number to mail out surveys typically being higher than on-line surveys. Hamilton (2010) produced a white paper that analyzed 199 surveys. The total response rate of these surveys, calculated using the total number of surveys sent out in the 199 surveys and the total number of responses for the 199 surveys, was 13.35 %. He noted that large invitation lists, >1,000, tend to be associated with lower individual response rates.

Viser et al. (1996) showed that surveys with lower response rates (near 20 %) tended to produce more accurate results than surveys with higher response rates (although it is likely that this could not be generalized). However, Holbrook et al. (2007) concluded that a low response rate does not necessarily equate to a lower level of accuracy but simply indicates a risk of lower accuracy.

Harris Interactive (2008), a well-established organization specializing in web-based surveys, used a convenience sample of 70,932 California residents in a survey of attitudes towards healthcare. An email was sent to potential respondents with a link to a web survey and non-respondents received one reminder email. The response rate for the Harris Interactive survey was 2 %.

Such response rates seem to be typical of on-line surveys of specialized populations. Similar surveys include the following: Stewart et al. (1992), a SCIENCEnet electronic survey received 118 responses from “a computer-based network…which has over 4,000 subscribers”(p. 2); the National Defense University Research Directorate Study (1978) based its conclusions on the responses from 21 experts; the Slade Survey (1990) based conclusions on responses from 21 respondents; the Global Environmental Change Report Survey (1990) had a response rate of approximately 20 % from a sample 1,500; the Science and Environmental Policy project (Singer 1991) received a 32 % response rate from a sample of 102, and later a 58 % response rate from another sample of 24.

Consequently the sampling method and the response rate for the survey of climate scientists do not appear distinct from other such undertakings. Questions were posed in the form of a 7 point Likert scale. Each extreme value (1 or 7) represents full subscription to the norm or counter-norm. Histograms are used to present the level of subscription to a norm or counter-norm. All participation was voluntary. Respondents were assured anonymity.

## Results: CUDO’s and Climate Science

### Communism Versus Solitariness

‘The scientist’s claim to ‘his’ intellectual ‘property’ is limited to that of recognition and esteem…’ (Merton 1942:273).^1^ Communism [often referred to as communality] implies that research results should be the property of the entire scientific community. Scientific findings constitute a common heritage in which the equity of the individual producer is severely limited.’ (Merton 1942:21). Ziman (1996:68) elaborates on communism stating ‘The norm of communalism requires that the fruits of academic science should be regarded as “public knowledge”. It thus covers the multitude of practices involved in the communication of research results to other scientists, to students, and to society at large.’ Solitariness, the counter-norm of communism, implies that findings should be kept secret at least until publication.

Among the questions of the survey, six dealt broadly with Communism–Solitariness:

1.1 Scientific results should be–private/public property
1.2 To hide information which might be of importance to other scientists is–totally acceptable/completely unacceptable
1.3 Other scientists should have free access to my data after I have published the initial findings–absolutely not/under all circumstances
1.4 I have the right to keep initial findings secret to ensure that I get full credit when the findings are published–absolutely/I have no rights at all
1.5 It is important to protect my individual scientific property rights–not at all/at all costs
1.6 How much are you in favour of sharing your research materials with your peers–not at all/100 % in favour.

**Fig. 1:**
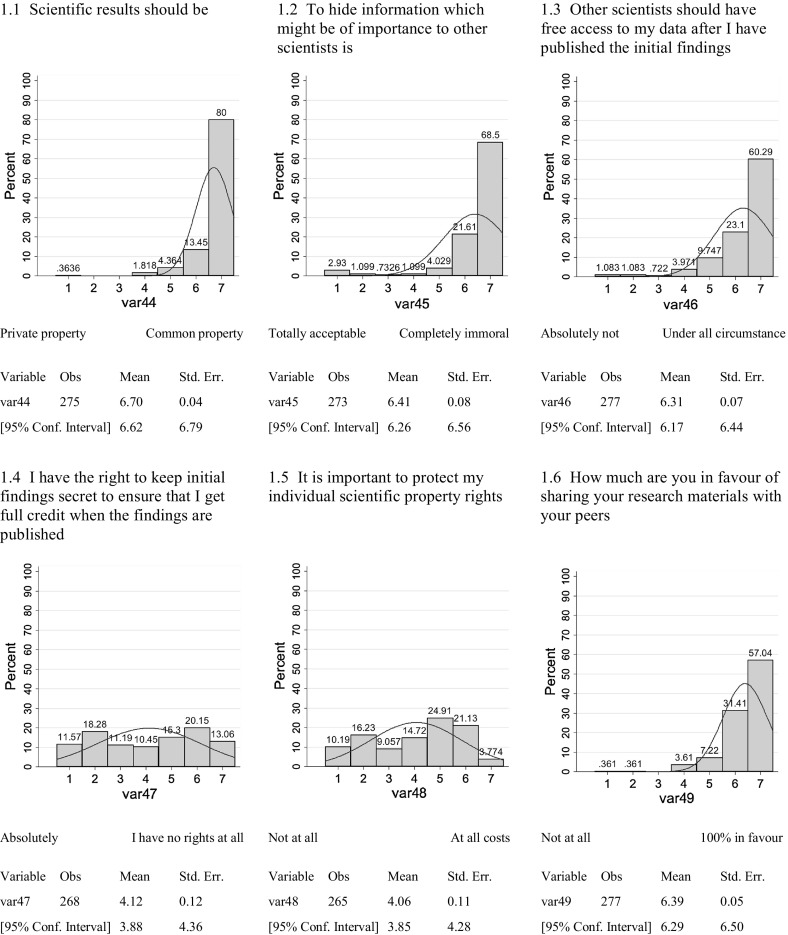
Communism versus Solitariness

The frequency distributions of responses on the 7-point Likert scale are shown in Fig. 1.

As the data in Fig. 1 indicates, in four out of six measures (1.1, 1.2, 1.3, 1.6) of the norm of communism, climate scientists held a high level of subscription to this Mertonian norm. Two measures (1.4, 1.5) show a less peaked response and are rather broad response patterns. In the 1.4 case, the demand for openness appears limited by the contemporary economy of science in which success is often defined by the first documenting of originality and novelty where primacy and publication lead to status and promotion.

However, in all measures used, there are at least some claims of full subscription to the norm. What appears to be the most questioned aspect of the norm of communism regards the timing at which information is released to the broader scientific community; i.e. after first publication.

### Universalism Versus Particularism

Universalism implies that scientific findings should be evaluated by ‘pre-established impersonal criteria: consonance with observations and with previously confirmed knowledge.’ Merton (1942:118). This implies that the reputation of an author should not enter an evaluation of his or her work. The definition is extended by Ziman (1996:68) to imply ‘that scientific propositions should be general enough to apply in any cultural context.’ Particularism, the counter-norm of universalism, suggests that a scientist might judge a contribution to science on the basis of the reputation of, or knowledge about, the author.

The survey addressed the issue of universalism/particularism using five questions:

2.1 Acceptance or rejection of scientific findings and claims should depend on personal feelings—never/always
2.2 People from outside of climate science disciplines should be allowed to contribute to climate science knowledge—not at all/very much
2.3 Obligation to publish findings even if they are contrary to your beliefs—never/always
2.4 Accept peer reviewed publications as accurate simply on the basis that they are published—never/always
2.5 Are well-known climate scientists, that are in agreement with findings similar to your own, perceived of as producing ‘better’ science than contributions made by unknown scientists?—never/always.

**Fig. 2:**
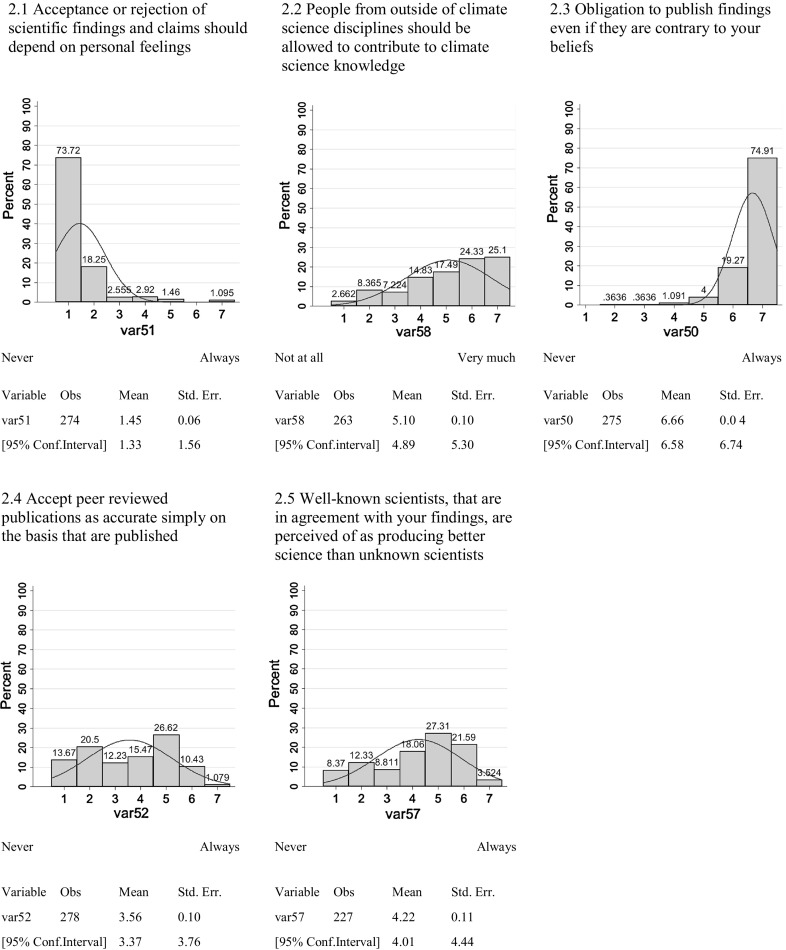
Universalism versus Particularism

Respondents to the survey demonstrated a high level of agreement with the following measures of the norm of universalism: (1) The obligation to publish findings even if they are contrary to personal beliefs (with no indication of full subscription to the counter-norm); (2) the acceptance or rejection of scientific findings should not depend on personal feelings, and; (3) that the scientific debate should be open to non-experts (Fig. 2).

### Disinterestedness Versus Interestedness

Disinterestedness implies that scientists should have no emotional or financial attachment to their work, be personally detached from truth claims, accept conclusions shaped only by evidence, and scientists should not campaign for a particular point of view or outcome. Disinterestedness also reflects the quality of perusing personal academic interests rather than the interests of funding agencies, policy priorities or institutional strategies. According to (Ziman 1996:68), ‘What it means is that they [scientists] in presenting their work publicly they must repress their natural enthusiasm for their own ideas, and adopt a neutral, impersonal stance.’ Interestedness, the counter-norm of disinterestedness, means that the scientist has personal interests at stake in the reception of his or her results and work. The survey included the following measures, indicating more the actual practice of science rather than what scientists perceive to be the ideals of science, but nonetheless, do address the ideals of science.

3.1 I try to ensure that my intellectual work is not influenced by my personal beliefs and values—never/always
3.2 I pursue research that is only of personal interest to me—never/always
3.3 How often do you have no choice but to align your research interests with funding opportunities—never/always
3.4 I feel there are unrealistic expectations (from sponsors/public/authorities) concerning the abilities of climate science—no expectations/always expectations.

There is a high level of full subscription to the norm of disinterestedness as indicated by claims that work is not influenced by personal beliefs and values (Fig. 3.1) and no full subscription to the counter-norm. In Fig. 3.1, most respondents claim to adhere to the norm of disinterestedness. However, measures 3.2–3.4 show a broad distribution of results with indications of full subscription to both the norm and counter-norm, with Fig. 3.3, that deals with the economy of science, demonstrating that the adherence to the counter-norm, interestedness, is much more evident.

**Fig. 3:**
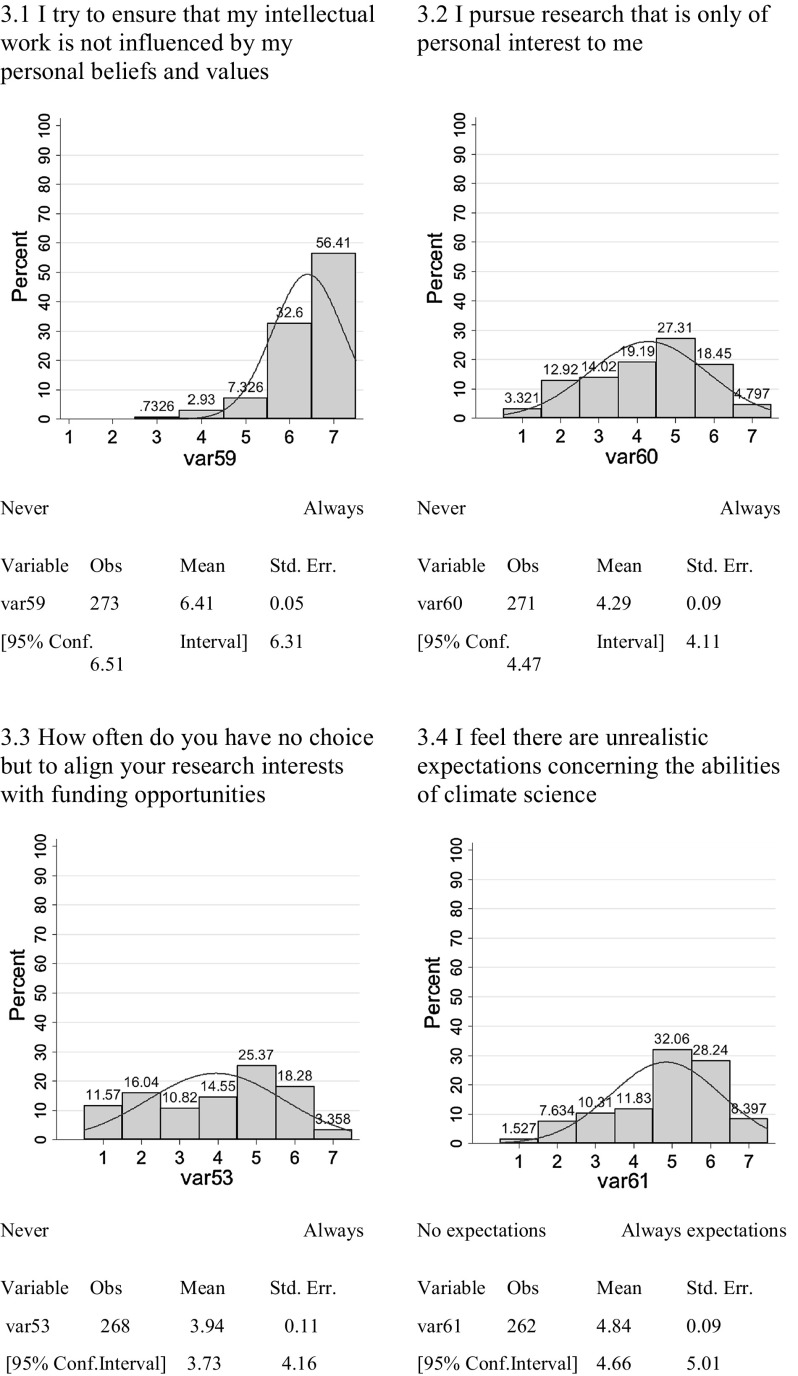
Disinterestedness versus Interestedness

### Organized Skepticism Versus Organized Dogmatism

Organized Skepticism implies that scientists should remain skeptical about research results and use caution in presenting premature conclusions that are tentative. Ziman (1996:68) adds,

[This] is the basis for many academic practices, such as carefully controlled critical controversy and peer review. … [It] merely stresses the constructive role of refutation as the natural partner of conjecture in the production of reliable knowledge. […] This social mechanism thus tests the claims of academic science in terms of rational qualities such as logical and factual consistency.

Organized dogmatism, the counter-norm for organized skepticism, is evident when a scientific career might rely on a particular premise being true, even if it creates a paradoxical situation when it comes to providing a scientific explanation. According to Zuckerman (1977:122) advocacy of any type runs contrary to the norm of organized skepticism, noting that ‘going to the lay public for legitimation and recognition violates the norm of organized skepticism since it by-passes the primacy of qualified peer appraisal.’ Dogmatism also includes scientists promoting their own most important findings, theories, or innovation.

Four of the questions in the survey deal with this norm:

4.1 I consider all new evidence, hypotheses, and theories, even those that challenge or contradict my work—never/always
4.2 I judge other contributions to my science on the basis of quality only—never/always
4.3 I assess the work of other scientists primarily on the status of the author—never/always
4.4 How often is there pressure to conform your research to fit with the findings of more prominent scientists—never/always.

In all four measures of the Mertonian norm of organized skepticism, full subscription to the norm considerably out-weights full subscription to the counter-norm of organized dogmatism. The majority of climate scientists responding to the survey claim to consider new material which may challenge their own claims (Fig. 4.1) and claim to consider these on the basis of the quality of the work only (Fig. 4.2). The claim is made that the author of the work is not an overly significant factor taken into consideration (Fig. 4.3) and the pressure to conform to the work of more prominent authors is not overly evident (Fig. 4.4).

**Fig. 4:**
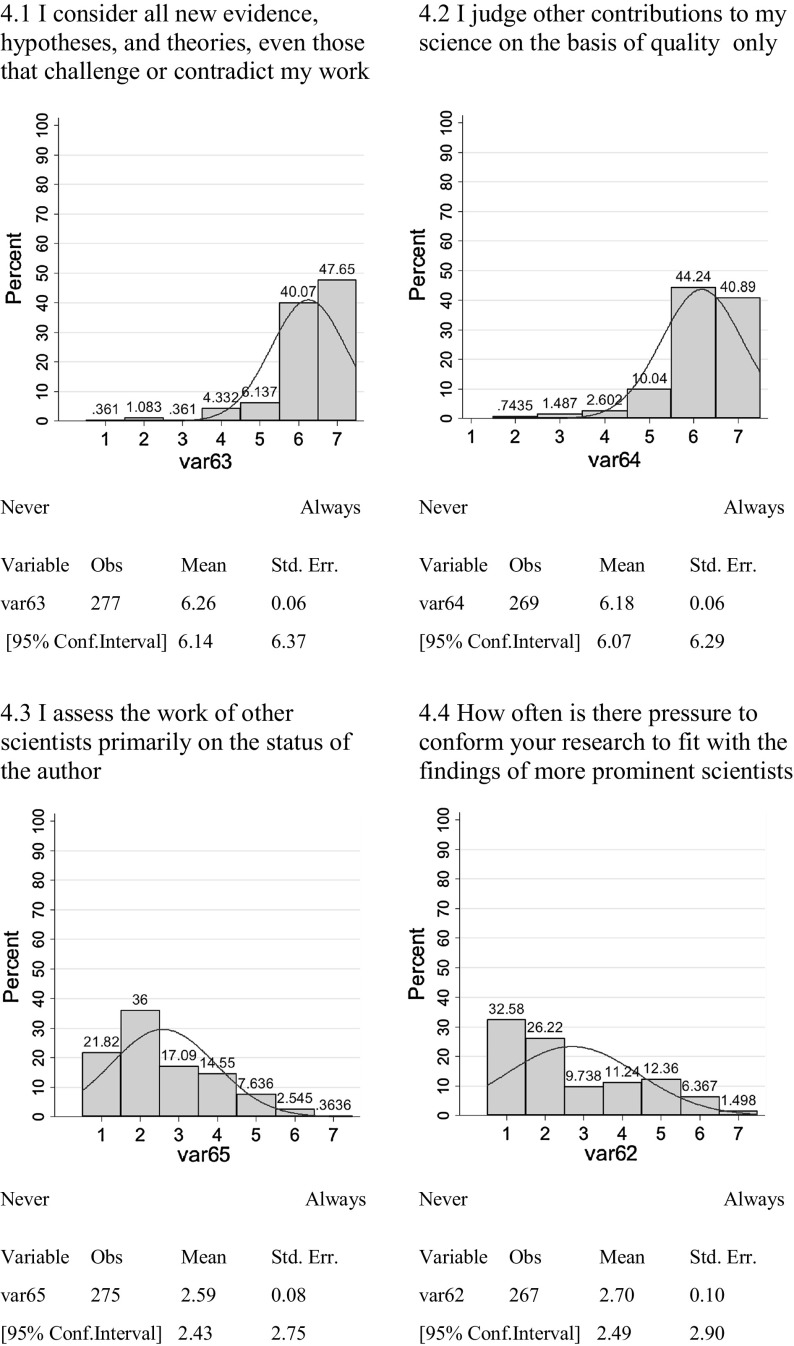
Organized Skepticism versus Dogmatism

## Discussion

Anderson et al. (2010), in the survey of NIH funded multidisciplinary scholars found:

1. there was greater levels of subscription to the norms than to counter-norms
2. in the physics/math/engineering sub-sample, adherence to the norm of communality was very high
3. in the physics/math/engineering sub-sample, adherence to the norm of universalism was high
4. in the physics/math/engineering sub-sample, adherence to the norm of disinterestedness was high in early career respondents but lower among mid-career respondents
5. in the physics/math/engineering sub-sample, adherence to the counter-norm of individualism was high in the mid-career respondents.

Macfarlane and Chen (2008), analyzing a multidisciplinary sample of UK academics concluded:

1. respondents tended towards a subscription to the counter-norm of secretiveness
2. 50 % of respondents claimed to be influenced by personal beliefs
3. disinterestedness was the least popular norm.
4. 42 % of respondents linked research to funding opportunities
5. 37 % of natural scientists supported the idea of expressing views in public
6. there was support for the norm of communism
7. there was strong support for the protection of individual property rights.

The 2013 survey of Bray and von storch (2014) was limited to only climate scientists. Given the sample, one would expect the physics/math/engineering sub-sample in the survey of Anderson et al. (2010) to be the most similar in terms of characteristics. The Bray and von Storch (2014) used multiple measures to capture each of Merton’s norms.

With reference to communism four of the six measures show a strong level of subscription to norm of communism. The weaker of the two measures address property rights, with more scientists claiming to subscribe to the counter-norm of individualism. This is consistent with the Anderson et al. (2010) finding that in the physics/math/engineering sub-sample, adherence to the counter-norm of norm of individualism was high in the mid-career respondents. In the 2013 Bray and von Storch survey, high and mid-career scientists constituted a large majority of the respondents, with only approximately 7 % of respondents reporting to have been involved in science for <5 years. Macfarlane and Chen (2008) found a similar tendency among a multidisciplinary sample of UK academics but did not provide any indication of the analysis of subgroups. Concerning the survey of climate scientists (Bray and von Storch 2014) overall, there was greater levels of subscription to the norm of communism than to counter-norm of individualism. This is consistent with both Anderson et al. (2010) and Macfarlane and Chen (2008), suggesting perhaps that given the character of contemporary science, it might be necessary to revise the features that characterize the Mertonian norms.

Concerning the norm of universalism, Anderson et al. (2010), in the physics/math/engineering sub-sample, found subscription to the norm of universalism to be high. Macfarlane and Chen (2008) did not comment on this norm. The Bray and von Storch (2014) survey again used multiple measures to assess the level of subscription to the norm of universalism. In two of the measures, subscription was extremely high, in two measures subscription was limited to a small majority while the blind acceptance of articles simply based simply on the fact that they are published received a rather broad range of responses with approximately 86 % of respondents claiming that, to some degree at least, they were willing to accept peer reviewed publications as accurate simply on the basis that they are published.

Considering the norm of disinterestedness, Anderson et al. (2010) observed that subscription was high in early career respondents but lower among mid-career respondents and according to Macfarlane and Chen (2008), disinterestedness was the least popular norm. The Bray and von Storch survey (2014) demonstrated mixed results. While personal beliefs and interests were claimed not to influence research, there were considerable claims that research interests were related to funding opportunities. The details of the measures used by Anderson et al. (2010) and Macfarlane and Chen (2008) are not readily available. However, it could be suggested that post-academic drivers (cf. Ziman 2000) are playing a significant role in re-shaping the norms of science. This implies the role of external forces is shaping contemporary science. EU framework funding programmes and similar structures elsewhere likely play a considerable role in the process (cf. Moriarty 2008). ‘Here the impact of a performative culture is linked to the need for a large number of academics to align their research interests with funding opportunities’. [Macfarlane and Cheng’s (2008:67)].

The last of the norms under analysis is the norm of organized skepticism. Neither Macfarlane and Chen (2008) nor Anderson et al. (2010) address the norm of organized skepticism. Data collected in the Bray and von Storch survey (2014) suggests there is a high level of subscription to this norm within the climate science community.

Merton’s CUDOs merely represent ideals for the conduct of science, norms which are not uncontested (e.g., Brysse et al. 2013). They offer a means to assess change and the normative shape of a scientific discipline under changing demands and conditions.

While the contemporary economy of science might contribute to the tendency of the counter-norm of communalism, it might also be that the moral content of the academic discipline plays an equal role, particularly when tangible rewards for scientists are fairly limited, as in the case of climate change science. Authors of claims contrary to the status quo, for example, are not greeted with healthy, skeptical curiosity but rather with disdain from the majority of peers. In such a case, it is probable that ‘secrecy’ and ‘property rights’ are adhered to for self-preservation as much as they are for self-gain.

Deviation from the norm of universalism might also be affiliated with the moral issues of the scientific claims. Climate science is divided into a number of opposing and/or disagreeing camps. At one extreme there are those who claim that risks of man-made climate change are immanent and potentially catastrophic. At the other extreme there are those scientists claiming the whole issue is a hoax. In the middle, there are those scientists who, although they accept that climate change is occurring [“manifestation” in Bray (2010)] and can hardly be explained without reference to elevated greenhouse gases (“attribution” in Bray 2010), but do not adhere to concepts of catastrophic climate change.

Disinterestedness, as noted above, implies that scientists should have no emotional or financial attachment to their work, be personally detached from truth claims, accept conclusions shaped only by evidence, and scientists should not campaign for a particular point of view or outcome. As the data indicated, in three of the four measures there was a broad spread of subscription to this norm. In one measure, however, addressing the extent to which personal values and beliefs influence intellectual work, there was a strong tendency of the respondents to claim that such beliefs and values did not shape their work. Given the immersion into a topic as morally charged as global warming, it is possible that the scientist becomes blinded to the forces shaping research interests. As Ziman (2000:31) pointed out, scientists can be ‘so immersed in their culture that the operating normative system is invisible to them’, consequently there is no reason the personal values that enter work would be detected by the individual. More recently, in the climate sciences, there are explicit calls for scientists to reject the norm of disinterestedness. On the matter of organized skepticism, that implies that scientists should remain skeptical about research results and have caution in presenting premature conclusions that are tentative.

The data suggests that while Merton’s CUDOs remain the overall guiding moral principles, they are not fully endorsed or present in the conduct of climate scientists: there is a tendency to withhold results until publication, there is the intention of maintaining property rights, there is external influence defining research and the tendency to assign the significance of authored work according to the status of the author rather than content of the paper. These are contrary to the norms of science as proposed by Robert K. Merton.

## Footnotes

1 In climate science the term *intellectual property* is not well-defined, and was hardly used prior to the public debates and legal conflicts, when opponents of publicly visible scientists demanded access to data and programs. In many cases, the scientists declined to provide this access arguing it was his or her intellectual property.
